# An Innovative Undergraduate Medical Curriculum Using Entrustable Professional Activities

**DOI:** 10.1177/23821205231164894

**Published:** 2023-04-24

**Authors:** Anne E Bremer, Marjolein H J van de Pol, Roland F J M Laan, Cornelia R M G Fluit

**Affiliations:** 1 Radboud Institute for Health Sciences, Department of Radboudumc Health Academy, 6029 Radboud University Medical Center, Nijmegen, the Netherlands; 2 Department of Primary and Community Care, Radboud University Medical Center, Nijmegen, the Netherlands; 3 Department of 6034 Radboudumc Health Academy, Radboud University Medical Center, Nijmegen, the Netherlands

**Keywords:** EPAs, clerkships, entrustment, feedback

## Abstract

The need to educate medical professionals in changing medical organizations has led to a revision of the Radboudumc's undergraduate medical curriculum. Entrustable professional activities (EPAs) were used as a learning tool to support participation and encourage feedback-seeking behavior, in order to offer students the best opportunities for growth. This paper describes the development of the Radboudumc's EPA-based Master's curriculum and how EPAs can facilitate continuity in learning in the clerkships. Four guiding principles were used to create a curriculum that offers possibilities for the students’ development: (1) working with EPAs, (2) establishing entrustment, (3) providing continuity in learning, and (4) organizing smooth transitions. The new curriculum was designed with the implementation of EPAs and an e-portfolio, based on these 4 principles. The authors found that the revised curriculum corresponds to daily practice in clerkships. Students used their e-portfolios throughout all clerkships, which stimulates feedback-seeking behavior. Moreover, EPAs promote continuity in learning while rotating clerkships every 1 to 2 months. This might encourage curriculum developers to use EPAs when aiming for greater continuity in the development of students. Future research needs to focus on the effect of EPAs on transitions across clerkships in order to further improve the undergraduate medical curriculum.

## Introduction

Health care worldwide is changing. We are dealing with more chronic illnesses,^1^ complex care, and multimorbidity, and care is increasingly being transferred to outpatient clinics and primary care. These changes call for medical professionals that have an extensive set of skills,^2,3^ both in medical proficiency and in other professional competencies, such as communication^4^ and organizational skills. Future medical doctors need to be able to adapt to different circumstances and to be devoted to lifelong learning. Educating medical professionals in these changing medical organizations requires a different kind of medical training. These developments have led to changes in medical education worldwide, which motivated the Radboud University's medical faculty to revise their undergraduate medical curriculum and its accompanying clerkship scheme.

**Figure 1.**
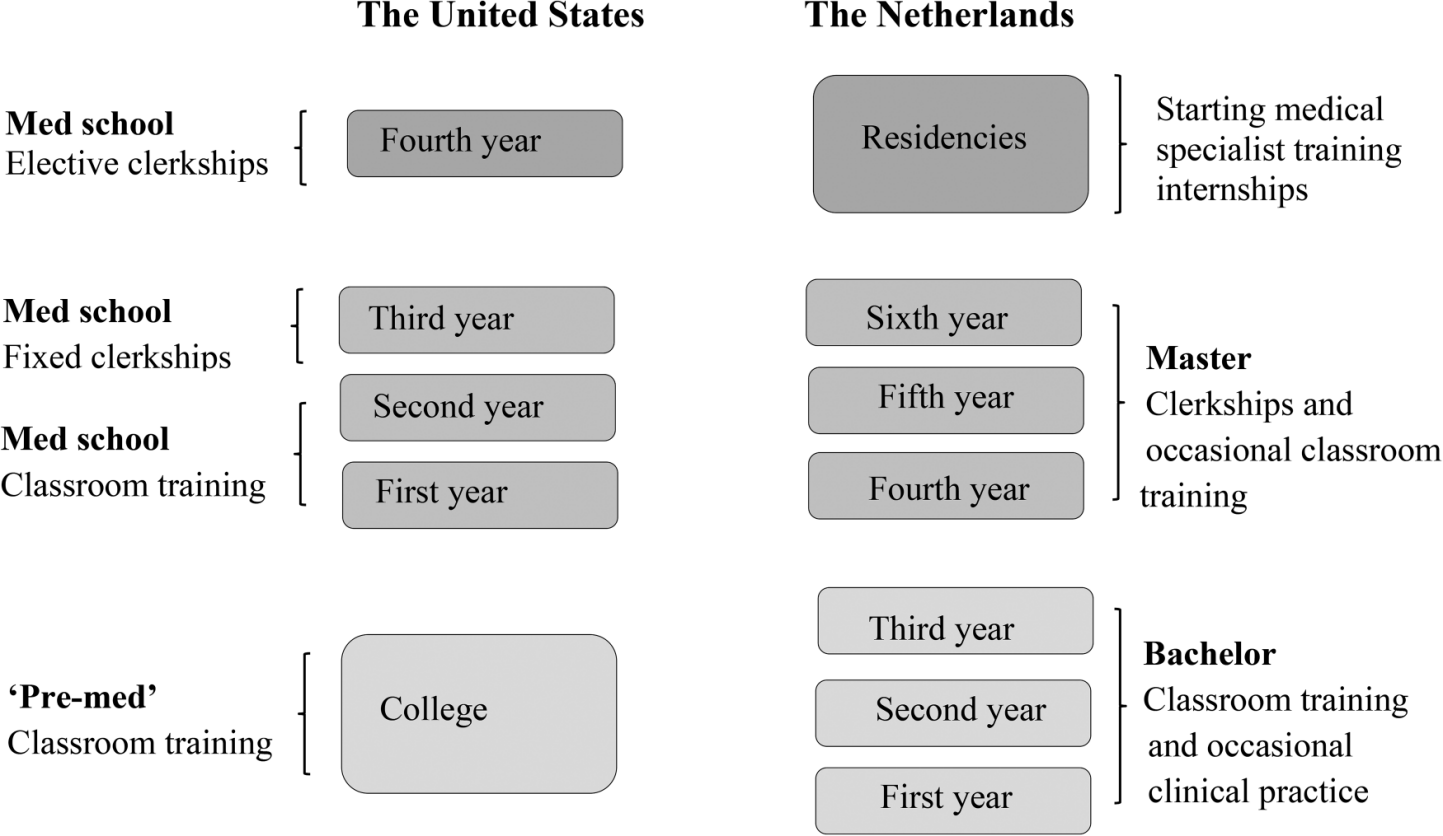
Undergraduate medical curricula of the United States and the Netherlands.

The Dutch undergraduate medical curriculum consists of 2 phases: the Bachelor's and the Master's phase, both taking 3 years. This is different from most international medical curricula, such as the undergraduate curriculum of the United States, which takes 4 years in total and is preceded by a preparatory program (pre-med). See Figure 1 for an overview of the differences between these curricula. The revision of the Radboud University's medical curriculum started in 2015 with the first 3 years of the undergraduate curriculum (the Bachelor's). The renewed Bachelor's curriculum stimulates professional development by focusing on self-regulated learning with more responsibilities for students, less classroom training, and more patient contact. We used the past 3 years to evaluate the implementation of the Bachelor's curriculum and collect ideas for revising the Master's curriculum (the clerkships).

Curriculum developers, supervisors in our undergraduate medical curriculum and medical students have, among others, been involved in the design and development of the revised master curriculum. In the Netherlands, Educational Law dictates to organize regular evaluation meetings with teachers, supervisors, and students (WHW Wet op het Hoger Onderwijs en Wetenschappelijk Onderzoek, Higher Education and Scientific Research Act). In the design and development process of our revised Master's curriculum, we organized several group interviews and discussions with involved staff and students, next to regular evaluation meetings, to get more insight into their desires for the new curriculum (Box 1). Furthermore, we based our new curriculum on the most recent academic standards. We intended to create a curriculum offering sufficient opportunities for personal and professional growth, and for this growth to be monitored. This would bring it in line with the renewed Bachelor's curriculum: both curricula are highly focused on professional development and lifelong learning, with smooth transitions from the Bachelor's all the way to residency.

**Box 1.** Ethical Approval and Evaluation Meetings.

Ethical approval:
The participants of our curriculum design process are not subjected to acts that are subject to the Medical Research Involving Human Subjects Act (WMO). On this basis, the Central Medical Ethical board region Arnhem-Nijmegen the Netherlands (CMO) declares that the curriculum design process does not fall under the remits of the WMO (Dossier number: CMO-020-6518). Subsequently, the curriculum design proposal was approved by the Education Board of the faculty of Medicine Nijmegen the Netherlands (Dossier number OMT2-180202). All participating students and teachers/coaches gave informed consent. All data are anonymized, no identifying information is present. The research and curriculum design process is conducted in accordance with the Netherlands Code of Conduct for Research Integrity (Netherlands Code of Conduct for Research Integrity | NWO).
Evaluation meetings:
As stipulated in the Higher Education and Scientific Research Act, every educational institution is required to organize evaluation meetings. For our curriculum design, some of these evaluation meetings have been extended and attendees have been asked to think and discuss about our curriculum design. The extension of these meetings was approved by the Education Board of the faculty of Medicine (see above). The extended evaluation meetings are minuted anonymously. The minutes are submitted to the attendees for approval. The input from these discussions has been used by our curriculum design team for the development of the curriculum (this article).

Regardless of how exactly a curriculum is constructed, medical education should be interprofessional and competency-based, instead of focusing only on skills and knowledge^5^ as we want to train medical doctors who are proficient in all the key competencies of medical health.^6^ To realize a Master's curriculum that would offer our students the best opportunity for professional and personal growth, we implemented entrustable professional activities (EPAs). The use of EPAs as a learning tool in undergraduate curricula is an important incentive for stimulating students’ professional growth, or professional identity formation, as they support participation in the medical department and encourage the students’ feedback-seeking behavior.^7^

Our EPAs are linked to a personal e-portfolio for recording daily feedback and monitoring professional growth. To familiarize our preceptors with the necessary knowledge and skills to work with the new EPA-based curriculum and the e-portfolio, all our senior supervisors have been trained intensively through both lectures and different exercises. For information about our training program, visit Digitaal portfolio – Radboudumc (in Dutch). Moreover, specifically trained student–teachers familiarized the staff of the affiliation hospitals with the new system. Furthermore, we created a teach-the-teacher: a strategy that focuses on strengthening and improving the didactic actions of the preceptor in order for them to organize a structured and effective learning environment for their scholars. This results in the ability of our affiliated hospitals to train their own preceptors.

In this paper, we describe the development of our EPA-based Master's curriculum and we discuss how EPAs can facilitate continuity in learning. We also explain how we integrated EPAs into the students’ daily work. By doing so, we hope to support other curriculum developers in realizing an EPA-based undergraduate medical curriculum.

## Guiding Principles for Professional Development

We used 4 guiding principles for professional development to create a curriculum that would offer the best possibilities for the development of our Master's students: (1) working with EPAs, (2) establishing entrustment, (3) providing continuity in learning, and (4) organizing smooth transitions. These principles will be explained here.

### Working With EPAs

The first principle we used for the development of the new Master's curriculum is working with EPAs. EPAs are “units of professional practice that can be fully entrusted to a trainee, as soon as he or she has demonstrated the necessary competence to execute this activity unsupervised.”^8^ EPAs provide a framework for experiencing professional practice in the workplace and for receiving direct feedback that can be used for professional development. Because of the students’ preference for immediate and direct feedback^9^ and the proven strength of this type of feedback,^10^ the use of EPAs, combined with short structured feedback moments, can be helpful in the professional development of medical students.

Although EPAs were initially developed for use in graduate medical education (GME), they are now also implemented in undergraduate medical education (UME).^11,12^ It seems a logical step to realize UME programs that use EPAs to prepare students for their residencies. Expectations of students’ professional clinical skills vary substantially among different (pre)clerkship departments.^13^ Residency program directors have shown concerns about the readiness of medical school graduates when they enter residency.^14,15^ Chen et al^11^ explained that with EPAs, expectations of the students’ skills are more explicit and descriptive, which helps both students and educators. Lomis et al^12^ added to this: “EPAs can serve as an outline for what a UME program must, at minimum, teach for a learner to be successful on day one of residency and what clinical supervisors in medical school must entrust their learners to be able to do without direct supervision in the next phase of training.” In this way, EPAs can bridge the gap between UME and GME.

### Establishing Entrustment

One of the key elements in the use of EPAs is entrustment; the second principle on which our curriculum is built. Trust between learners and supervisors is crucial for medical education.^16^ Trust, however, is not the same as entrustment: where trust is considered to be someone's willingness to be vulnerable to someone else's actions without being able to control the other person, entrustment is about assigning responsibility to another person.^17^ Schumacher et al^17^ explained that trust is a state of mind, whereas entrustment is an act, and “trust is necessary but not sufficient to make an entrustment decision.” A supervisor might consider a student trustworthy but still decide to perform the task him- or herself because of certain context-related factors, such as a patient's specific illness or the perceived risk.^17^ This clearly separates trust from entrustment.^18,19^

If students receive sufficient support and trust from professionals in the workplace, this makes it easier for them to express their uncertainties and to ask questions, which promotes learning.^20^ In order to earn formal entrustment, students must have opportunities to gain experience with increasing responsibility and ad hoc entrustment decisions.^21^ It is recommended that entrustment decisions are based on longitudinal observations of the students’ performance with students actively participating in decision-making processes.^22^

### Organizing Continuity in Learning

The third principle we used for the development of our Master's curriculum is organizing continuity in learning, meaning that students can carry on with their learning process without constantly having to start over again. Out of all phases in medical education, clerkships in particular offer many opportunities to train broadly educated doctors as they provide students with important clinical practice. Students develop a broader perspective on various aspects of medical care and experience a diversity of medical settings in a variety of hospitals.

The traditional model of medical education is organized around block clerkships (BCs), where students spend several weeks in the same medical department but rotate regularly, which might result in a lack of clinical participation in the workplace^23^ and difficulties with their development in practice.^24^ In contrast to BCs,^25^ an important trend in UME is the implementation of longitudinal integrated clerkships (LICs).^6,26,27^ Students in LICs spend more time at a certain medical department, which provides them with more opportunities for building relationships^28^ and with continuity in learning.^29^ Seeing patients over time allows students to build relationships with patients and thus to gain a deeper understanding of diseases.^24^

Continuity in learning can be achieved not only in LICs but also in BCs. Hirsch et al^27^ distinguish between 4 different types of continuity: continuity in care, supervision, curriculum, and idealism. Continuity in care allows students to be involved with patients over longer periods of time. Continuity in supervision implies that all educators and students involved are connected within the learning community, and that educators and faculty are engaged in transparent conversations about patient care, with secured time for teaching and faculty. Continuity in curriculum can be achieved when the medical curriculum forms an integrated program with space for self-reflection and critical thinking. The most essential form of continuity, however, is continuity in idealism. To sustain the idealistic values with which students start medical school, they need relevant contact with patients (care), possibilities to self-reflect (curriculum), and iterative dialogue about medical practice (supervision). “In this way, the entire learning community nurtures and maintains a spirit of idealism—idealism that will surely be translated into enhanced learning, greater patient satisfaction.”^27^

Our revised undergraduate curriculum is based on the BC structure, where we aimed to realize continuity in our current system in the most optimal way. We decided to implement EPAs in order to stimulate continuity in learning by focusing on the long-term development of skills. In this situation, EPAs can be used to support longitudinal assessment, even when students regularly rotate clerkships^30^ as students are encouraged to transfer their learning goals and experiences to the next clerkship, where new supervisors have insight into the process as well. This allows for continuity in supervision: EPAs provide transparent dialogue and students and supervisors are always connected. With EPAs and the e-portfolio, students have proof of all they have done and learned before, which allows them to take their learning to the next level in the subsequent clerkship. In this way, our curriculum forms an integrated whole with plenty of space for self-reflection (continuity in curriculum). Furthermore, our curriculum leaves more room for continuity in learning as students do not need to start all over again at the launch of each new clerkship. All in all, we believe that, with this curriculum, we offer the right foundation for facilitating the continuity of idealism as well.

### Providing Smooth Transitions

The fourth principle for the development of our Master's curriculum focuses on providing smooth transitions for our students. As we have seen before, starting over again is a common phenomenon for medical students in BCs as they frequently transition between different workplaces.^31^ After each transition, students have to acclimatize to the new culture, environment, and customs of that particular medical department. These transitions allow students to grow,^32^ both personally and professionally, but they can also cause problems in their development as they have to reexamine who they are.^33^ We consider the transitions between clerkships to be smoother and less complicated for our students, as they keep track of their own development by recording the feedback they received and monitor their progression with EPAs in longitudinal e-portfolios. In this way, they obtain written “proof” of their experiences and growth in their personal e-portfolios, which is insightful for themselves as well as for their supervisors. To inspire other curriculum developers to facilitate continuity in learning in a block-oriented clerkship curriculum, we will describe the structure and the underlying principles of our curriculum in this paper.

## The Revised Undergraduate Medical Curriculum

### The Use of EPAs for Continuity in Learning

The Radboud University Medical Center's Master's curriculum was revised in 2019. We decided to use some specific EPAs that all students have to achieve at the end of their programs as the main components of our curriculum. These EPAs ought to stimulate learning and asking for feedback and foster transitions between clerkships. Like most universities in the Netherlands, we have a long history of BC clerkships in the Master's curriculum. We did not change the rotation structure of our BC-oriented Master's curriculum, but we invested in EPAs to promote continuity of learning. As explained before, EPAs support medical students in carrying learning goals over to their next clerkship, with stimulates learning over a longer timeframe. Using EPAs reinforces the lifelong learning ambition of the Radboudumc, as they provide connections between all 3 phases (Bachelor's, Master's, and residency) in medical education.

Our new EPA-based Master's curriculum is also more in line with the renewed Bachelor's curriculum, where students have their first encounters with medical practice. Upon entering the Master's curriculum, students have been spending considerable time in practice, which prepares them well for the clerkships. This makes them more experienced in their first clerkships than students from the former Bachelor's curriculum: they know how to work independently, to ask feedback regularly, and to set learning goals. This is why the new Master's curriculum had to be equipped with more practical experience for medical students in order to endorse their learning goals. Learning goals differ from person to person, and in an EPA-based curriculum students are able to design their own path, in line with their individual qualities and interests. Furthermore, we realized that the previous Master's curriculum offered too few opportunities for specific feedback and insufficient preparation for residency. In the new Master's curriculum, the continuous development of their learning goals is likely to be a good preparation for independent practice.

Moreover, the Radboudumc highly values the development of cross-cutting skills for its medical students. Skills that cut across different fields, such as communication, ethics, and teamwork, are becoming increasingly important.^3^ These skills were formerly called “soft” skills, but regardless of their precise definition, “they describe skills that are not discipline or career specific, that can and should be developed within educational settings, and that prepare individuals for lifelong learning in a rapidly changing global world.”^3^ Developing cross-cutting skills do not come naturally; they are learned over time when working with different people in various teams.^34^ Besides developing these skills, the EPA-based curriculum provides students with opportunities for gaining both medical and nonmedical experience. With the use of EPAs, students gradually are more and more entrusted to perform activities that correspond with their abilities.

### The EPA-Based Master's Curriculum Structure

Our Master's curriculum is founded on the following EPAs, all including sub-EPAs: (1) medical consultation, (2) medical procedures, (3) guidance and education, (4) communication and collaboration, and (5) nonclinical activities. EPAs 1 to 4 include specific activities that all medical students have to accomplish by the end of their clerkships. For EPA 5, although not an EPA in the strict sense of the word, we expect students to actively request feedback on a specific, individually chosen, professional activity. The feedback process of EPA 5 is comparable to the process of the other EPAs. Every EPA has a detailed description of (1) the activity itself, (2) for which students are declared competent, and (3) what knowledge, skills, and attitudes students need to master to achieve the EPA at a certain level. For an overview of what EPA 1 and its sub-EPAs look like, see Box 2. All EPAs can be found in Appendix 1.

Furthermore, medical students are required to formulate a personal training plan with learning goals and individual development objectives. They request feedback from their supervisors and other staff on a daily basis. This feedback is written down in an online feedback form, called a KPB (mini clinical evaluation exercise [CEX] or feedback assessment). All KPBs are collected in a personal e-portfolio (part of the personal training plan), which provides an overview of the activities students are entrusted to perform and their professional development. Supervisors have to provide narrative feedback along with an entrustment-supervision scale grade on a specific EPA-related task. Although our current research was ongoing, we made some changes to the curriculum. Previously, we expected supervisors to provide prospective feedback, but this appeared to hinder the content of the feedback and the feedback process. Our students needed to explain the feedback process very often, as supervisors were used to providing retrospective feedback. This is why we are now working with retrospective feedback based on a specific EPA, accompanied by a certain supervision level (see Box 3). Our supervisors considered this way of providing feedback more instinctive and straightforward.

**Box 2. table2-23821205231164894:** EPA 1: Medical Consultation.

1.1 History taking and physical examination
1.2 Formulating differential diagnosis
1.3 Formulating plan of investigation
1.4 Interpreting results of common diagnostic tests
1.5 Formulate treatment plan

**Box 3.** Supervision Levels.

The student showed to master the activity to the following extent:	
1a	I (supervisor) performed the activity together with the student.
2	The student performed the activity independently, but my (supervisor's) presence and additions were necessary.
After oral discussion adjacent to the activity:	
3a	I (supervisor) had to review the activity completely and I made necessary additions.
3b	I (supervisor) had to review specific elements of the activity, and I made a few additions.
3c	I (supervisor) did not have to review the activity (but did it anyway as I have the final responsibility).
4	The student performed the activity independently and we discussed our findings later.

### The Ins and Outs of Feedback in Our Curriculum

The narrative feedback needs to present an evaluation of both positive aspects (“what is going well?”) and aspects that need further improvement (“what needs more attention”?). Supervisors are expected to build on earlier feedback and judgments from colleagues. This way of supervising resembles the supervision of residents, who also bring EPAs they obtained earlier into their next workplace. Feedback on all EPAs is transferred to the next clerkship. EPA 1 and EPA 5 operate slightly differently than the other EPAs: for EPA 1, only general history taking is taken into other clerkships but not the profession-specific sub-EPAs, and for EPA 5, the feedback level is not relevant given the individual nature of the activities. The entrustment-supervision scale shows at what level a student is expected to perform a particular activity in the future (eg, supervision level 3a indicates that the supervisor must check every aspect of this activity in the future). See [Table table2-23821205231164894]Box 3 for an overview of all supervision levels.

Because the e-portfolio makes it possible to access all EPAs achieved thus far, the individual competencies of EPAs 2 to 4 are transferred to the next clerkship(s). This allows supervisors to review the development of their students longitudinally rather than during one specific clerkship only, which prevents redundant repetition of activities students already master, and it allows them to perpetuate or develop certain clinical skills even further. EPAs are used for (1) performing assessments in the workplace, (2) tracking the development of students with e-portfolios, and (3) preparing a personal training plan. At the end of the Master's phase, every student needs to have achieved all EPAs at certain, predetermined, level.

When a student receives 5 feedback assessments at the same level for a specific EPA, we assume that he or she masters this individual professional activity at that level. The received feedback level also guides other supervisors in deciding what responsibilities they can entrust to students when performing a certain activity. In this way, EPAs and feedback assessments are used as an entrustment tool. More importantly, however, as working with EPAs and feedback assessments provide us with loads of valuable narrative feedback of every individual student, this gives us great input for the performance reviews of our students at the end of every clerkship, in which supervisors determine whether a student has passed the clerkship or not. EPAs and feedback assessments serve as the basic ingredients for our evaluations.

## Discussion

This paper describes our first experiences with an EPA-based Master's curriculum. We prepare broadly trained medical doctors,^6^ as EPAs guide the students’ extensive development in different professional areas. Our curriculum and the e-portfolios correspond to daily practice in clerkships. Students use their e-portfolios throughout all their clerkships, which stimulate feedback.^7^ EPAs support students in choosing appropriate learning goals and tasks,^7^ which allow them to grow and gain proficiency in a wide range of medical procedures and knowledge. This corresponds with our first principle for professional development, which is “working with entrustable professional activities.”

Furthermore, our curriculum acknowledges the fact that entrustment is one of the key factors in the successful implementation of EPAs,^16^ corresponding with our second principle, “establishing entrustment.” Students take their earlier feedback into their next clerkship, where new supervisors are expected to build on this feedback to decide about appropriate responsibilities for a particular student. These decisions indicate important moments of trust for a student on a daily basis. This new feedback routine is a great strength of our curriculum as feedback moments take place every day and are vital for learning. This abundance of feedback moments is expected to greatly advance the building of trust.

However, trust is also an important topic of further consideration. From earlier research, we know that “distrust is at the core of hierarchical systems.”^35^ Supervisors trust students not only on the basis of direct observations but also on observations from some distance.^36^ In our curriculum, supervisors are expected to entrust students based on feedback from colleagues, which could be considered “observations from some distance.” We are not yet aware of how supervisors experience this new feedback routinely and whether they rely on their colleagues enough to base their own trust on a colleague's feedback. In a study on trusting residents, Hauer and colleagues describe that “supervisors expressed ambivalence about the value of the often very general prior information they held or received about residents.”^37^ Duitsman and colleagues add to this by explaining that supervisors rarely use feedback from an assessment tool and tend to rely more on opinions of faculty members and their own beliefs.^38^ More research is needed on trust and entrustment between supervisors and undergraduate students in their clerkships.

Our new Master's curriculum, moreover, offers students continuity in learning, representing our third principle for professional development, “organizing continuity of learning.” We see that our students bring their learning goals to their next clerkship, where they develop further and complete their learning goals. Within our BC structure, we show that EPAs are a way to promote continuity in learning while rotating clerkships every 1 to 2 months, which might encourage other curriculum developers to use EPAs when aiming for greater continuity in the development of students.

We realized that changing the Master's curriculum would be more difficult than the Bachelor's curriculum, as the former involves working with many affiliations. With clerkships taking place at different hospitals and other institutions, all staff involved have to be aware of and participate in the innovations. We turned this weakness into a strength by introducing the e-portfolios: as students take these to every new clerkship, supervisors are always able to see what students have achieved before. This creates clarity for all institutions, supervisors, and students themselves and facilitates gentle transitions between clerkships, which is in line with our fourth principle for professional development, “providing smooth transitions.”

Students also indicated that they had a significant impact on this process as they taught supervisors how to work with this new system. In the first few months, supervisors appeared to be struggling with the implementation of EPAs, which gave our students the (unintended) opportunity to explain and demonstrate the procedures of the new system. In this way, students operated as change agents in our new curriculum.

### Strengths and Limitations

The major strength of this study is that the guiding principles for professional development can be used by other curriculum developers who wish to implement an EPA-based undergraduate medical curriculum. The principles give clear directions on how to use EPAs in block rotations and how they can facilitate continuity in learning.

We acknowledge that this study also has a few limitations. Firstly, medical students will never be completely entrusted to perform an activity unsupervised, as this level of supervision will only be achieved in residency. EPAs do support medical students with a more structured framework for learning and preparation for residency, but the current system does have the limitation of not being able to fully entrust the medical student. This makes it difficult to entirely understand and implement the concept of entrustment. However, it is very useful that the longitudinal e-portfolio provides preceptors of the following block rotation insight into what a student has done and achieved in earlier block rotations. A second limitation is that, for this study, we did not collect a lot of evaluation data, as our main intention is to present other curriculum developers with a framework on how to work with EPAs in undergraduate medical curricula. However, both experienced preceptors and medical students were involved in the design and development of our curriculum (see Box 1). In earlier research, we do show first results of how an EPA-based curriculum supports the development of medical students,^7^ and we will be presenting more results in further research.

### Future Directions

Over the past few years, we have gained useful knowledge and experience with EPAs in the undergraduate curriculum, in which EPAs appeared to be valuable for professional identity formation and feedback-seeking behavior.^7^ This has provided us with tools to further develop the Master's program into one with a longitudinal character and greater continuity, whether this is continuity of learning, supervision, curriculum, or idealism.

## Conclusion

We developed this EPA-based medical Master's curriculum for medical students at the Radboudumc in Nijmegen, the Netherlands. Our curriculum promotes continuity in learning for students and provides excellent opportunities for feedback. Our first experiences support this design, but we need to acquire more knowledge of the effect of EPAs on transitions taking place between clerkships, and the process of gaining and giving trust by supervisors. Currently, we are investigating a better understanding of EPAs across clinical rotations, as this is needed.

## Supplemental Material

sj-docx-1-mde-10.1177_23821205231164894 - Supplemental material for An Innovative Undergraduate Medical Curriculum Using Entrustable Professional Activities Click here for additional data file. Supplemental material, sj-docx-1-mde-10.1177_23821205231164894 for An Innovative Undergraduate Medical Curriculum Using Entrustable Professional Activities by Anne E Bremer, Marjolein H J van de Pol, Roland F J M Laan, and Cornelia R M G Fluit in Journal of Medical Education and Curricular Development
