# Supplementary material for: An Innovative Undergraduate Medical Curriculum Using Entrustable Professional Activities
Source: J Med Educ Curric Dev. 2023 Apr 24;10:23821205231164894. doi: 10.1177/23821205231164894 (PMC10134152; doi:10.1177/23821205231164894)
Supplement: sj-docx-1-mde-10.1177_23821205231164894 - Supplemental material for An Innovative Undergraduate Medical Curriculum Using Entrustable Professional Activities [file sj-docx-1-mde-10.1177_23821205231164894.docx]

## **Appendix 1: EPAs in the Radboudumc**

| **EPA** | **Sub-EPA** |
| --- | --- |
| 1. Medical consultation | - 1. History taking and physical examination   2. Formulating differential diagnosis   3. Formulating plan of investigation   4. Interpreting results of common diagnostic tests   5. Formulating treatment plan |
| 1. Medical procedures | 2.1 Participating in the operating room  2.2 Rectal examination, inserting catheter  2.3 Common profession-specific activities  2.4 Installing a drip, venous puncture, injections  2.5 Basic life support, Automated External Defibrillator |
| 1. Guidance and education | 3.1 Discussing diagnostic and therapeutic possibilities  3.2 Discussing results and prognosis  3.3 Difficult or unusual conversations  3.4 Providing information on healthy lifestyle and disease prevention  3.5 Motivational interviewing |
| 1. Communication and collaboration | 4.1 Presenting oral and written reports that document a clinical encounter  4.2 Inter- and intraprofessional collaboration |
| 1. Non-clinical activities | This is not a clearly defined EPA; students can record any non-clinical activity. |
